# Limb salvage with tibial nerve repair via acute shortening and gradual lengthening in a Gustilo type IIIB tibial shaft fracture: EMG-proven reinnervation of intrinsic foot muscles

**DOI:** 10.1016/j.tcr.2025.101288

**Published:** 2025-11-17

**Authors:** Shunsuke Sato, Satoshi Hatashita, Michiyuki Hakozaki, Takuya Kameda, Yoichi Kaneuchi, Masayuki Ito, Yoshihiro Matsumoto

**Affiliations:** a Department of Traumatology and Reconstructive Surgery, Fukushima Medical University School of Medicine, 1 Hikarigaoka, Fukushima-shi, Fukushima, 960-1295, Japan; b Traumatology and Reconstructive Surgery Center, Aizu Chuo Hospital, 1-1 Tsuruga-machi, Aizuwakamatsu, Fukushima, 965-0011, Japan; c Department of Orthopaedic Surgery, Fukushima Medical University School of Medicine, 1 Hikarigaoka, Fukushima-shi, Fukushima, 960-1295, Japan

**Keywords:** Severe open tibial fracture, Acute shortening and gradual lengthening, Tibial nerve rupture, Electromyography

## Abstract

Limb-length discrepancy following lower-extremity trauma significantly affects patients' functioning and quality of life and is generally avoided by anatomical reconstruction. When lower-extremity trauma is accompanied by tibial-nerve injury, standard treatment protocols may require modifications. We describe a 38-year-old Japanese man with a Gustilo type IIIB open tibial-shaft fracture and complete tibial-nerve rupture, which we treated with acute shortening and gradual lengthening (ASGL). The tibia was shortened by 40 mm to facilitate the tibial nerve's direct end-to-end neurorrhaphy at a healthy site. Initial stabilization was achieved using an external fixator, followed by staged internal fixation and soft-tissue reconstruction with a free latissimus dorsi flap. Once flap integration was confirmed, gradual lengthening with an Ilizarov external fixator restored the limb length, with no length discrepancy. Two years post-injury, the patient regained protective plantar sensation and full weight-bearing ability. He achieved advanced functional milestones: a single-leg stance and hopping. Electromyography (EMG) confirmed the intrinsic foot-muscle reinnervation, including the abductor hallucis and interosseous muscles. The patient resumed snowboarding. This case demonstrates that ASGL has advantages for skeletal and soft-tissue reconstruction and nerve recovery optimization. Although nerve grafting can restore sensation, motor recovery is often limited. In young patients with a complete tibial nerve rupture, tibial shortening to allow direct primary repair may provide superior functional outcomes in both sensory and motor domains. This appears to be the first documentation by EMG of the functional recovery of intrinsic foot muscles following tibial-nerve repair.

## Introduction

A discrepancy in the length of the two limbs after an individual experiences lower-extremity trauma can lead to a variety of complications including lower back pain, joint pain in the lower extremities, secondary osteoarthritic changes, postural instability, impaired balance, and alterations in gait mechanics [1]. In the management of severe open tibial fractures with segmental bone loss, it is therefore essential to reconstruct the bone length anatomically. Treatment strategies for this purpose are selected based on the morphology and size of the defect and may include the Masquelet technique, free vascularized bone grafting, or bone transport using external fixation [[2], [3], [4]]. However, in cases of open tibial fractures accompanied by tibial nerve injury, standard protocols such as the so-called “fixation for fractures (fix) and flap” may not always be applicable [5]. We report a case of an open tibial shaft fracture with complete tibial nerve rupture in which acute shortening and gradual lengthening (ASGL) was used to enable reliable end-to-end nerve repair. This approach resulted in not only the restoration of plantar sensation but also meaningful recovery of the patient's intrinsic foot muscle function.

## Case presentation

**Fig. 1:**
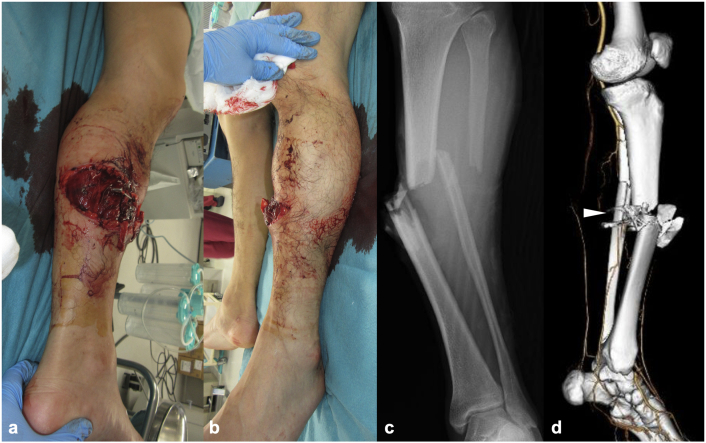
The initial clinical and radiographic findings at presentation of the patient, a 38-year-old male. (a) Medial-posterior view of the left lower leg. (b) Anterolateral view of the left lower leg. (c) Anteroposterior plain radiograph of the left lower leg showing a diaphyseal tibial fracture. (d) Three-dimensional CT angiography of the tibia and surrounding vasculature. The posterior tibial artery was interrupted at the level of the tibial fracture (*(white arrowhead*).

The patient was a 38-year-old Japanese man who sustained an injury after striking his left lower leg against a metal pole while landing from a snowboard jump. He was transported to our hospital by an ambulance the same day. On the initial examination, a deformity of the left lower leg and a 20-cm transverse open wound on the medial aspect of the mid-shaft were noted (Fig. 1a,b). The dorsalis pedis artery was palpable bilaterally, but the posterior tibial artery was not palpable, and a complete loss of sensation on the plantar surface was observed.

**Fig. 2:**
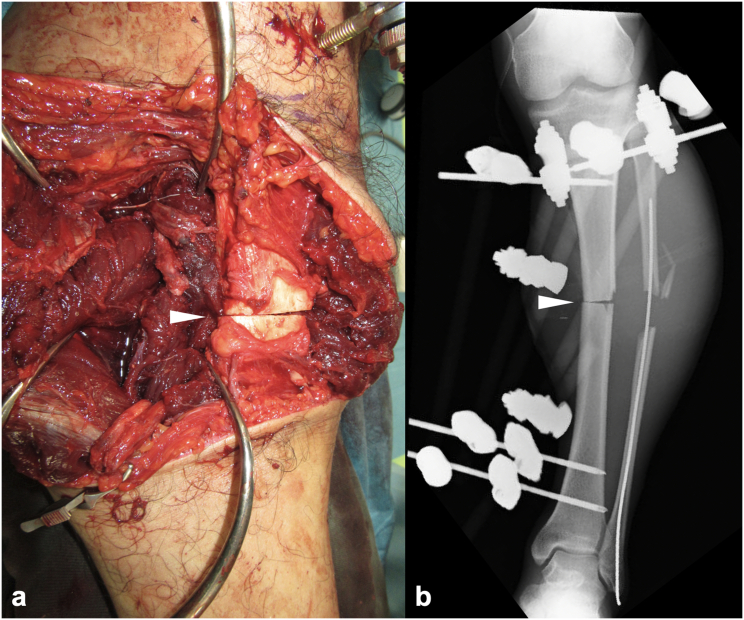
Intraoperative tibial osteotomy and shortening. (a) Intraoperative photograph showing the approx. 4 cm of tibial shortening, stabilized using a modular external fixator (*(white arrowhead*). (b) Post-shortening anteroposterior plain radiograph showing bony contact between fragments (*(white arrowhead*), achieved through external fixation.

**Fig. 3:**
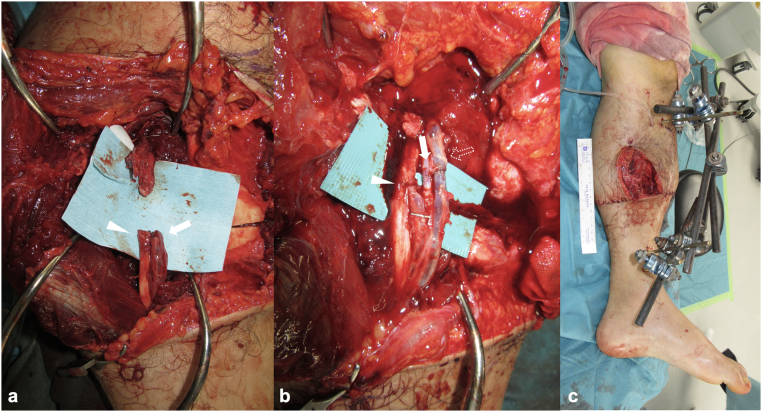
Intraoperative and immediate postoperative findings. (a) The freshened tibial nerve (*(white arrowhead*), posterior tibial artery, and vein (*(white arrow*). (b) The end-to-end repair of the tibial nerve (*(white arrowhead*), posterior tibial artery (*(white arrow*), and posterior tibial vein (*white dotted arrow*) in the healthy stumps. (c) Medial view of the left lower leg immediately after surgery. Primary wound closure was not feasible because of muscle bulging following the shortening and the need for additional incisions.

Plain radiographs revealed tibial and fibular shaft fractures (AO/OTA classification: 42B3b and 4F2Ab) (Fig. 1c), and contrast-enhanced computed tomography (CT) demonstrated disruption of the posterior tibial artery at the level of the tibial fracture (Fig. 1d). Based on the diagnosis of a left Gustilo type IIIB open tibial shaft fracture, posterior tibial artery injury, and tibial nerve injury, emergency surgery was performed on the day of the injury. The open wound was irrigated and debrided, and the injury was assessed. A complete transection of the posterior tibial artery and vein as well as the tibial nerve was observed at the fracture site. The tibial nerve was debrided until healthy tissue was reached, but a direct end-to-end repair was not feasible. The tibia was thus acutely shortened (by 40 mm) at the site of the wedge fragment, and a modular-type external fixator was applied (Fig. 2), enabling end-to-end neurorrhaphy of the tibial nerve (Fig. 3a,b).

The posterior tibial artery and vein were repaired using end-to-end anastomosis (Fig. 3a,b). Due to additional proximal skin incisions along the posteromedial tibial axis for nerve identification and debridement, as well as muscle bulging from the bone shortening, the wound could not be closed over a 9 × 6-cm area (Fig. 3c). The wound was temporarily managed with negative pressure wound therapy (NPWT), and the initial surgery was completed.

**Fig. 4:**
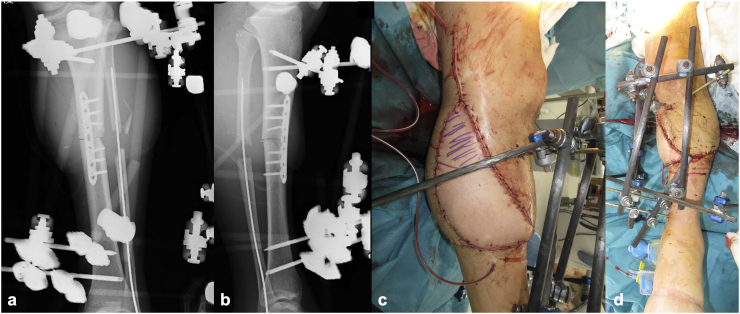
Postoperative radiographic and clinical findings following the ‘Fix and Flap’ technique. (a) Anteroposterior and (b) lateral plain radiographs of the left lower leg after internal fixation. (c) Medial and (d) lateral clinical views showing reconstruction with a free latissimus dorsi musculocutaneous flap.

On postoperative day 4, a second-stage ‘fix and flap’ procedure was performed. Internal fixation using a small limited-contact locking compression plate (LC-LCP) plate (Johnson & Johnson, Somerville, NJ, USA) was applied to the fracture site while the external fixator was retained (Fig. 4a,b). Soft-tissue reconstruction was performed using a free latissimus dorsi musculocutaneous flap, with the thoracodorsal artery anastomosed to the popliteal artery in an end-to-side fashion and the thoracodorsal vein anastomosed to the popliteal vein in an end-to-end fashion (Fig. 4c,d).

**Fig. 5:**
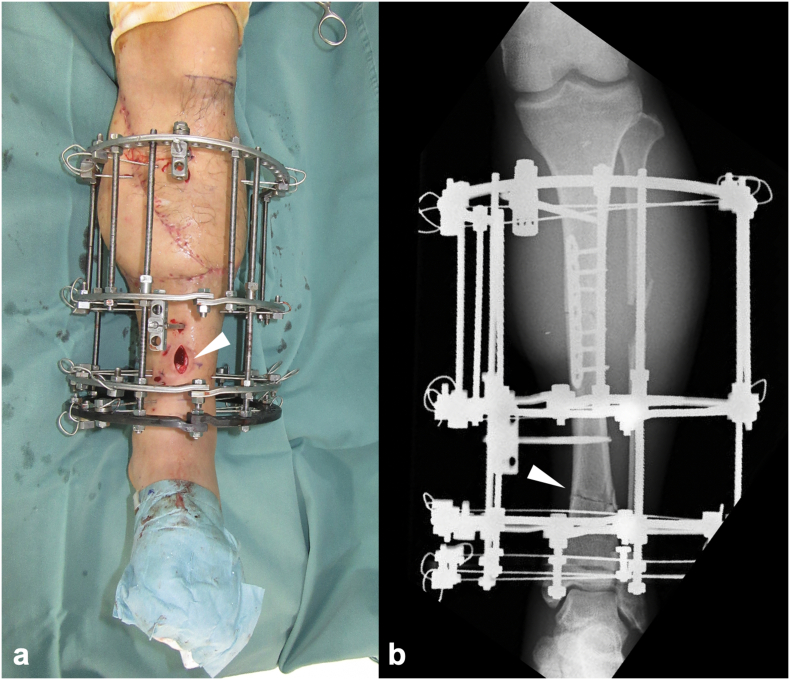
Findings following the application of the Ilizarov external fixator. (a) Intraoperative photograph showing the osteotomy site through a small incision (*(white arrowhead*). (b) Anteroposterior plain radiograph showing the osteotomy at the distal diaphysis of the tibia (*(white arrowhead*).

**Fig. 6:**
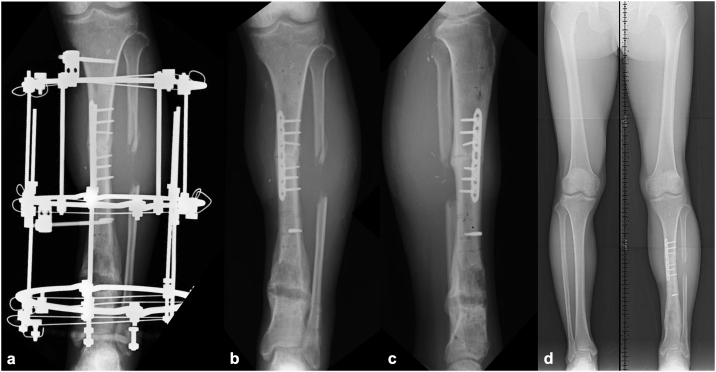
Radiographic findings after the limb lengthening. (a) Anteroposterior radiograph at 94 days after the application of the Ilizarov external fixator (IEF). (b) Anteroposterior and (c) lateral radiographs at 196 days (post-IEF removal). (d) Weight-bearing anteroposterior radiograph of both lower limbs 6 months post-injury showing no evident leg-length discrepancy.

On day 41 post-injury, once the flap's stability was confirmed, the modular-type fixator was converted to an Ilizarov external fixator (IEF), and an osteotomy was performed at the distal diaphysis to avoid the vascular pedicle of the flap. Bone lengthening was initiated 48 days post-injury (Fig. 5). On day 102 post-injury (61 days after the IEF placement), approx. 30-mm bone lengthening was achieved, with radiographic evidence of callus formation. A second osteotomy was performed on day 116 (75 days after the IEF placement). Bone lengthening was completed at 40 mm by day 135 (94 days post-IEF placement) (Fig. 6a), and the IEF was removed on day 237 (196 days after IEF placement) (Fig. 6b,c). The external fixation index was 49 days/cm.

**Fig. 7:**
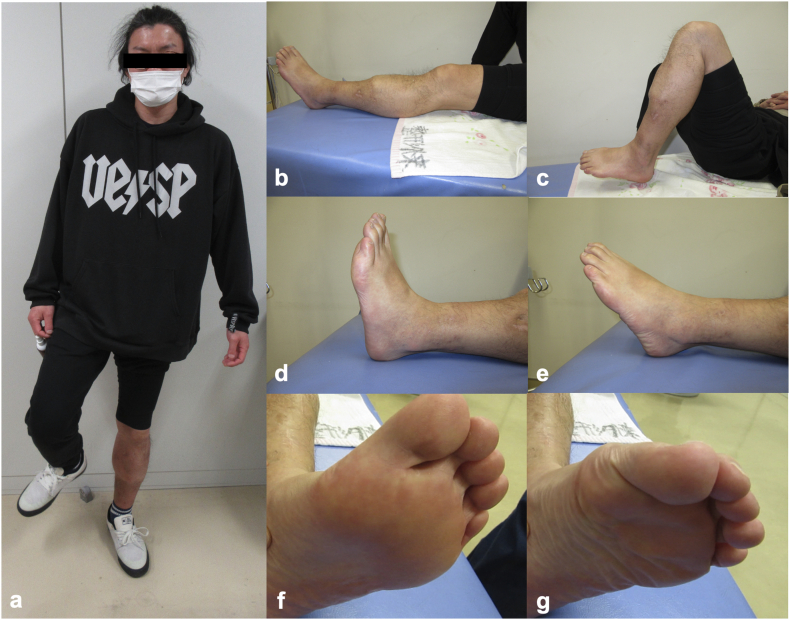
The patient's clinical functioning 2 years after the injury. (a) Single-leg stance on the affected limb. (b) Full knee extension and (c) knee flexion. (d) Ankle dorsiflexion and (e) plantarflexion. (f) Active toe extension and (g) toe flexion including the interphalangeal joint of the hallux.

During the patient's postoperative course, a pin-site infection developed but was successfully controlled with wound care and systemic antibiotics administered both intravenously and orally. At ∼6 months post-injury, the patient was able to bear full weight and exhibited no leg length discrepancy (Fig. 6d). At 12 months post-injury, he had no pain and had returned to work with independent ambulation. At 2 years, he was capable of a single-leg stance (Fig. 7a) and jumping, with a knee range of motion of 0° extension and 135° flexion and ankle dorsiflexion and plantarflexion of 20° and 25°, respectively (Fig. 7b–e). Toe extension and flexion were possible, including active flexion of the interphalangeal joint of the hallux (Fig. 7f,g). Semmes-Weinstein monofilament testing revealed protective sensation recovery: purple (4.17) at the forefoot and midfoot (contralateral: 4.08), and red (4.56) at the hindfoot (contralateral: 4.17).

**Fig. 8:**
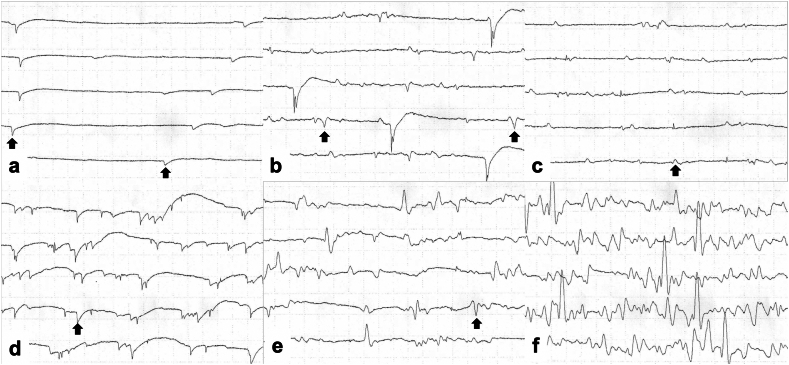
Electromyographic findings of intrinsic foot muscles at 2 years post-injury. a–c: Abductor hallucis muscle. d–f: Interosseous foot muscles. Scale: vertical = 200 μV/division, horizontal = 10 ms/division. (a) Positive sharp waves (PSWs) at rest (*black arrow*) indicate denervation. (b) Motor unit potentials (MUPs) during mild contractions (*black arrow*), suggesting voluntary reinnervation. (c) During strong contraction, the MUPs increased, but the interference pattern remained insufficient. (d) Clear PSWs in the interosseous muscles at rest (*black arrow*), indicating denervation. (e) MUPs were evident during mild contractions (*black arrow*). (f) A dense interference pattern during strong contractions, indicating good motor recovery.

At 2 years post-injury, electromyography (EMG) (Neuropack X1, MEB-2300 series; Nihon Kohden, Japan) of the patient's foot showed positive sharp waves (PSWs) and motor unit potentials (MUPs) in the interosseous muscles and partially in the abductor hallucis, indicating reinnervation of the intrinsic foot muscles innervated by the tibial nerve (Fig. 8). The patient's Lower Extremity Functional Scale score was 69, and he was able to snowboard again.

## Discussion

A key consideration in the treatment of this patient was whether tibial shortening was necessary to facilitate end-to-end neurorrhaphy of the tibial nerve. If the primary objective was limited to restoring plantar sensation, an alternative strategy could have involved early bone fixation to preserve limb length — likely using an intramedullary nail in this case — soft-tissue reconstruction with flap coverage, and tibial-nerve reconstruction through nerve grafting. This approach might significantly reduce the overall treatment period and allow for earlier weight-bearing ambulation. In addition, if neither bone shortening nor additional skin incisions were performed, the flap surgery itself could potentially have been avoided.

However, Soucacos et al. reported that among 46 cases of Gustilo type IIIB/IIIC extremity injuries, four patients with tibial nerve injury underwent nerve grafting, which enabled the recovery of protective plantar sensation and ambulation; nonetheless, they experienced recurrent plantar ulceration [6]. Similarly, Halms et al. described the case of an 18-year-old patient with a severe open tibial fracture and tibial nerve defect treated with a 10-cm sural nerve graft. Although the patient regained protective sensation, thermal and pain sensations, and coarse touch on the sole, ambulation still required the use of a cane, indicating that a tibial nerve injury can significantly affect gait quality [7].

Conversely, a report summarizing the cases of 135 patients with tibial nerve injuries revealed that in cases with fresh lacerations, primary neurorrhaphy performed within 72 h resulted in ≥Grade 3 recovery in 100 % of the patients. This outcome was significantly superior to the 86 % recovery rate observed with secondary nerve grafting [8]. These findings indicate that to achieve more reliable functional recovery of the tibial nerve after a traumatic rupture, freshening of the nerve ends and performing direct end-to-end repair at a healthy site is preferable.

Parmaksizoglu et al. described 13 cases of traumatic lower-leg amputation or Gustilo type IIIC open tibial fractures treated with acute shortening and early or delayed lengthening [9]. They noted that in eight of these cases, end-to-end neurorrhaphy was performed for a tibial nerve rupture, and all eight procedures provided restoration of plantar sensation and independent ambulation [9]. In that study, the protective sensation was considered to be adequately restored if the patient could detect a 6.0-g monofilament (red: 4.74) as part of the Semmes-Weinstein (SW) test [9]. Other reports have defined protective sensation as the detection of a 5.07 monofilament (also red) on the SW scale [10]. Based on these standards, our patient's case — with SW values of 4.17 at the forefoot/midfoot and 4.56 at the hindfoot — can be considered to have achieved sufficient restoration of plantar protective sensation. However, our search of the relevant literature did not identify detailed reports that specifically evaluated the recovery of motor innervation following tibial nerve repair in the context of lower-leg trauma.

Tibial nerve injury in the lower leg not only results in sensory disturbance of the plantar surface; it also causes dysfunction of the intrinsic foot muscles, leading to impaired propulsion during gait and diminished support of the foot arch [11]. Intrinsic foot muscles such as the abductor hallucis and flexor digitorum brevis function to attenuate the impact in the early stance phase and enhance plantar rigidity during the terminal stance phase, thereby contributing to a stable gait [12]. Riddick et al. emphasized that these intrinsic muscles are essential not only for normal walking but also for modulating energy absorption and generation in the foot during non-steady-state movements, such as stair ascent/descent and acceleration/deceleration in sports activities [12]. The intrinsic foot muscles thus play critical roles in supporting the foot arch and ensuring dynamic stability under loads.

Akuzawa et al. used EMG to evaluate the activation of intrinsic foot muscles during gait, and they reported that the abductor hallucis and quadratus plantae were activated at an earlier time point compared to the extrinsic muscles and maintained activity until the late propulsion phase [13].

In our patient's case, the tibial shortening by 40 mm enabled the direct end-to-end repair of the tibial nerve, resulting in the restoration of protective plantar sensation. EMG revealed signs of reinnervation in the abductor hallucis and interosseous muscles, i.e., PSWs and MUPs. The patient also regained advanced dynamic lower-limb functions, including single-leg stance and hopping, which indicates that his functional recovery extended beyond sensory restoration to include the motor function of the intrinsic foot muscles.

Artificial nerve conduits have been described as a reconstruction option for peripheral nerve injuries. Shirakura et al. reported a tibial nerve injury at the ankle in a 15-year-old boy, in which a 16-mm defect was repaired using two 3-mm-diameter artificial conduits (Nerbridge®, Toyobo, Osaka, Japan) placed in parallel [14]. At 3 years and 9 months postoperatively, the protective sensation was restored, and motor nerve conduction testing showed favorable outcomes, indicating the potential utility of artificial conduits for defects <20 mm in length. However, the inner diameter of such conduits is limited by manufacturing constraints, and for large-caliber nerves such as the tibial nerve (≥4 mm in dia.), a parallel use of multiple conduits may be required. Moreover, the diffusion of oxygen and nutrients within artificial conduits is limited, and the regenerative efficiency may decline in nerve gaps that are >20 mm. The quality of regeneration may thus be inferior compared to that provided by autologous nerve grafting or nerve transfers [14,15].

Taken together, our findings suggest that, especially in younger patients, performing tibial shortening to enable secure the end-to-end repair of a damaged tibial nerve at a healthy site may be a rational and effective strategy for optimizing the patient's postoperative motor function and gait quality.

## Conclusion

We have provided the case details of a Gustilo type IIIB open tibial shaft fracture with complete tibial nerve rupture treated with ASGL. To the best of our knowledge, this is the first report to demonstrate functional recovery of intrinsic foot muscles following tibial nerve repair in such a setting, as evidenced by EMG findings. Although the prolonged treatment duration is a limitation of this approach, ASGL may be a viable option, particularly in younger patients, not only to restore plantar sensation but also to achieve the functional recovery of intrinsic foot muscles, thereby improving the long-term quality of activities of daily living.

## CRediT authorship contribution statement

**Shunsuke Sato:** Conceptualization, Data curation, Writing – original draft, Writing – review & editing. **Satoshi Hatashita:** Supervision, Writing – review & editing. **Michiyuki Hakozaki:** Supervision, Writing – review & editing. **Takuya Kameda:** Supervision, Writing – review & editing. **Yoichi Kaneuchi:** Writing – review & editing, Supervision. **Masayuki Ito:** Supervision. **Yoshihiro Matsumoto:** Supervision.

## Informed consent

The patient was informed that data from the case would be submitted or publication and gave their consent.

## Consent for publication

All authors approved.

## Ethical approval

Ethical approval for this retrospective study was obtained from the Ethics Committee of Fukushima Medical University (Ref. No. General 30082).

## Ethical statement

Informed consent for publication was obtained from the patient, and this report was conducted in compliance with the tenets of the Declaration of Helsinki.

## Declaration of Generative AI and AI-assisted technologies in the writing process

During the preparation of this work, the author(s) used ChatGPT (OpenAI) solely for the purpose of checking grammar and improving language clarity. The scientific content, structure, and conclusions of the manuscript were entirely written and developed by the author(s), who take full responsibility for the content of the publication.

## Declaration of competing interest

The authors have no conflicts of interest to disclose and received no external sources of funding.
